# The Role of Lactic Acid on Wound Healing, Cell Growth, Cell Cycle Kinetics, and Gene Expression of Cultured Junctional Epithelium Cells in the Pathophysiology of Periodontal Disease

**DOI:** 10.3390/pathogens10111507

**Published:** 2021-11-18

**Authors:** Taichi Ishikawa, Daisuke Sasaki, Ryo Aizawa, Matsuo Yamamoto, Takashi Yaegashi, Tarou Irié, Minoru Sasaki

**Affiliations:** 1 Division of Molecular Microbiology, Department of Microbiology, Iwate Medical University, 1-1-1 Idai-dori, Yahaba-Cho, Morioka 028-3694, Japan; msasaki@iwate-med.ac.jp; 2 Division of Periodontology, Department of Conservative Dentistry, School of Dentistry, Iwate Medical University, 1-3-27 Chuo-dori, Morioka 020-8505, Japan; daisukes@iwate-med.ac.jp (D.S.); tyaegasi@iwate-med.ac.jp (T.Y.); 3 Department of Periodontology, School of Dentistry, Showa University, 2-1-1 Kitasenzoku, Ohta-ku, Tokyo 145-8515, Japan; r-aizawa@dent.showa-u.ac.jp (R.A.); yamamoto-m@dent.showa-u.ac.jp (M.Y.); 4 Division of Anatomical and Cellular Pathology, Department of Pathology, Iwate Medical University, 1-1-1 Idai-dori, Yahaba-Cho, Morioka 028-3694, Japan; tarou@iwate-med.ac.jp

**Keywords:** cell cycle, junctional epithelium, lactic acid, periodontal disease, short-chain fatty acids

## Abstract

Lactic acid (LA) is short-chain fatty acid, such as butyric acid and propionic acid, that is produced as a metabolite of lactic acid bacteria, including periodontopathic bacteria. These short-chain fatty acids have positive effects on human health but can also have negative effects, such as the promotion of periodontal disease (PD), which is caused by periodontal pathogens present in the gingival sulcus. PD is characterized by apical migration of junctional epithelium, deepening of pockets, and alveolar bone loss. Thus, the junctional epithelial cells that form the bottom of the gingival sulcus are extremely important in investigating the pathophysiology of PD. The aim of this study was to investigate the effect of LA on wound healing, cell growth, cell cycle kinetics, and gene expression of cultured junctional epithelium cells. The results showed that stimulation with 10 mM LA slowed wound healing of the junctional epithelial cell layer and arrested the cell cycle in the G0/G1 (early cell cycle) phase, thereby inhibiting cell growth. However, cell destruction was not observed. LA also enhanced mRNA expression of integrin α5, interleukin (IL)-6, IL-8, intercellular adhesion molecule-1, and receptor activator of nuclear factor kappa-B ligand. The results of this study suggest that stimulation of junctional epithelial cells with high concentrations of LA could exacerbate PD, similarly to butyric acid and propionic acid.

## 1. Introduction

It is well known that lactic acid bacteria (LABs), as probiotics, convey beneficial effects to human health by inhibition of pathogenic microorganisms, immune regulation, antiallergic activities, and antagonism [1,2]. For example, LABs are reported to enhance host immunity and prevent many diseases, including cancer [3], as well as produce bacteriocin, which strongly inhibits the growth and proliferation of *Streptococcus mutans*, the major causative agent of caries formation, and *Porphylomonas gingivalis*, a periodontal pathogen [4]. In addition, LAB, which includes *Actinomyces*, *Bacteroides*, *Lactobacillus*, and *Fusobacterium*, reside in the intestinal tract and oral cavity [5,6]. However, lactic acid (LA), a metabolite of LABs, promotes caries formation [7], and high concentrations in the blood and cerebrospinal fluid can damage tissues and nerves, ultimately resulting in death [8,9,10]. Thus, depending on location, LABs can have both beneficial and detrimental effects on the host [11]. LA is a short-chain fatty acid, sharing biochemical characteristics with butyric acid, propionic acid, acetic acid, and formic acid, that can promote and prolong gingival inflammation [6,12]. In vitro experiments have confirmed that butyric acid and propionic acid are involved in the adhesion, growth, cell cycle, and protein synthesis of gingival fibroblasts [13]. The gingival sulcus of the periodontitis patient frequently exhibits local butyric, propionic, and LA concentrations of approximately 1 to 30 mM [14]. At low concentrations, LA slows apoptosis of polymorphonuclear leukocytes and has an opposite effect at high concentrations [14]. Butyric acid, in particular, is associated with periodontitis [15,16,17] but is also reported to have beneficial health effects, such as suppression of colitis via the induction of regulatory T cell differentiation, especially in the intestinal tract [18,19]. Thus, like butyric acid, LA can have adverse effects depending on the site and concentration, although the detailed association with periodontal disease (PD) remains unclear.

PD is caused by bacteria that form dental plaques in the gingival sulcus, such as periodontal pathogenic bacteria and oral streptococci, in association with pathogenic factors produced by these plaque bacteria, such as protease and fimbriae, and the host immune response [20,21,22]. Clinically, it is characterized by apical migration of epithelium (junctional epithelium) along the surface of the root to the root tip, loss of attachment, deepening of pockets, and loss of alveolar bone [23]. Short-chain fatty acids, including LA, are produced in dental plaque with increased concentrations in the gingival crevicular fluid of patients with severe periodontitis as compared to healthy controls [24,25]. However, the effects of LA on PD, as opposed to butyric acid and propionic acid, remain unclear. In addition, the junctional epithelium has different properties from gingival fibroblasts and other cells used in previous studies of PD [26,27]. Therefore, the aim of the present study was to investigate the effects of LA on wound healing, cell growth, cell cycle kinetics, and gene expression of cultured junctional epithelium (JE-1) cells, a newly established junctional epithelial cell line [28]. This study will be useful for elucidating the pathophysiology of periodontal disease and could be utilized in future clinical practice.

## 2. Results

### 2.1. LA Inhibited Cell Mobility and Proliferation

**Figure 1:**
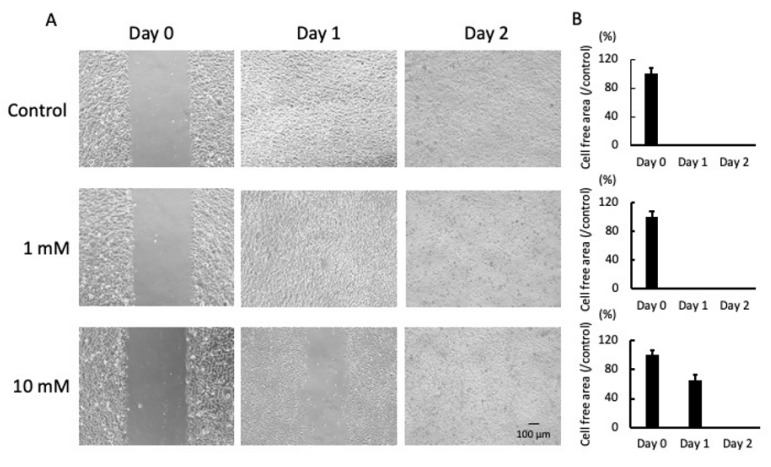
Photomicrographs of the scratch assay on days 0, 1, and 2 (**A**). Cells were incubated in fresh medium supplemented with 1 or 10 mM LA. Representative photomicrographs of four independent experiments are shown. The cell-free areas were measured every 24 h from day 0 to day 2 (**B**). Values are presented as the mean ± SD of four independent experiments. Scale bar = 100 µm.

As shown in Figure 1A, cells migrated to the cell-free area and completely filled the gap after 1 day (control and 1 mM LA), while the gap of the cells cultured with 10 mM LA remained until day 2. Under all culture conditions, relatively few dead and floating cells were observed on day 2. In the group treated with 10 mM LA, the cell-free area on day 1 was approximately 65% of that on day 0 (Figure 1B).

### 2.2. LA Affected Cell Proliferation

**Figure 2:**
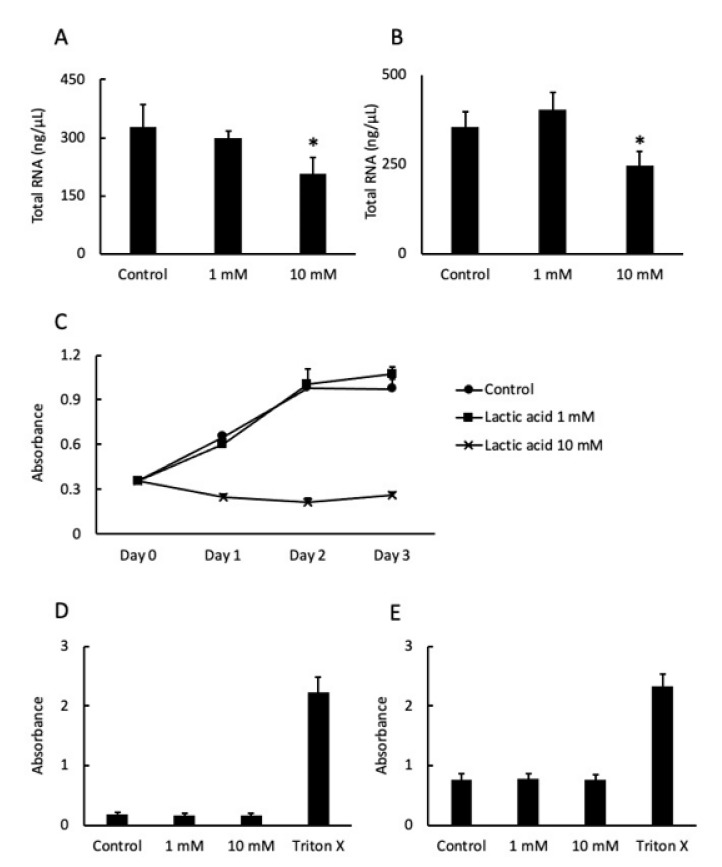
The cells were cultured in fresh medium supplemented with 1 or 10 mM LA or fresh medium only (as a control). The bars indicate the amount of total RNA extracted from the cultures at 24 h (**A**) and 48 h (**B**). Values are presented as the mean ± SD of six independent experiments. Cell proliferation was assessed by measuring absorbance. (●), (■), and (×) represent the control, 1 mM LA, and 10 mM LA groups, respectively (**C**). Values are presented as the mean ± SD of eight independent experiments. Cell viability was measured in terms of LDH activity at 24 h (**D**) and 48 h (**E**). An 1% Triton X 100 was used to measure the maximum enzyme activity. * *p* < 0.05 vs. control cells (0 mM). Values are presented as the mean ± SD of five independent experiments.

The amount of total RNA extracted from the cells cultured with 10 mM LA was significantly reduced after 24 and 48 h compared to the respective controls (Figure 2A,B). Cell proliferation was completely inhibited by 10 mM LA from day 1 to day 3 (Figure 2C). However, there was no difference in LDH activity at 24 and 48 h, apart from the group treated with Triton X 100, which destroyed all cells (Figure 2D). LDH activity at 48 h was slightly higher than at 24 h (Figure 2C,D).

### 2.3. LA Interrupted Progression of the Cell Cycle

**Figure 3:**
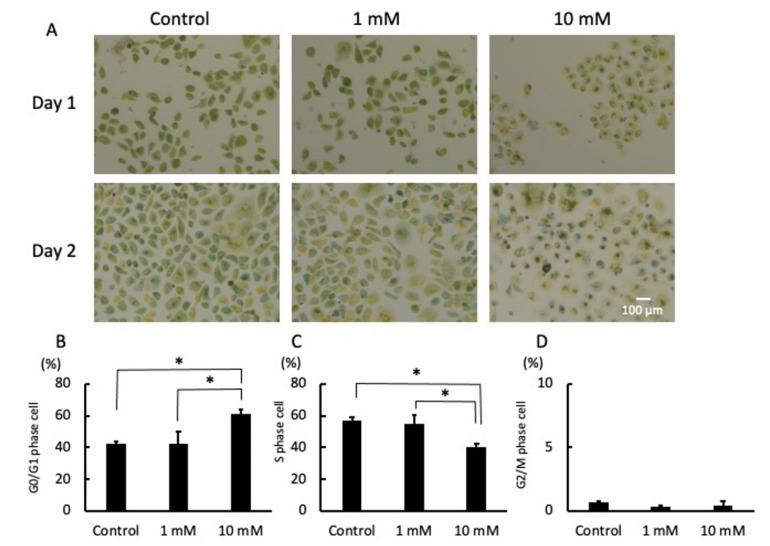
Photomicrographs of the cell cycle on days 1 and 2 (**A**). The cells were cultured with fresh medium supplemented with 1 or 10 mM LA or fresh medium only (as a control). Representative photomicrographs of four independent experiments are shown. The phase of the cell cycle was determined by color pixel analysis. The percentages of cells in the G0/G1 phase (**B**), S phase (**C**), and G2/M phase (**D**) are shown. * *p* < 0.05. Values are presented as the mean ± SD of four independent experiments. Scale bar = 100 µm.

There was no significant difference in the morphology or number of cells cultured with 1 mM LA for 24 h as compared to the control. However, when cultured with 10 mM LA for 24 h, there was an inhibition in the proliferation of cells and most were small and round (Figure 3A). In addition, the characteristics were remarkable, and the gap was relatively wide among the cells cultured with 10 mM LA for 48 h (Figure 3A). The results of color pixel analysis showed that a significantly large proportion of cells cultured with 10 mM LA for 24 h were in the G0/G1 phase (yellow color) (Figure 3B). In contrast, relatively few cells cultured with 1 mM LA and those in the control group (0 mM) were in the S phase (green color) (Figure 3C). There was no significant difference in the relatively low proportion of cells in the G2/M phase cells (blue color) among the groups (Figure 3D).

### 2.4. LA Influenced Gene Expression

**Figure 4:**
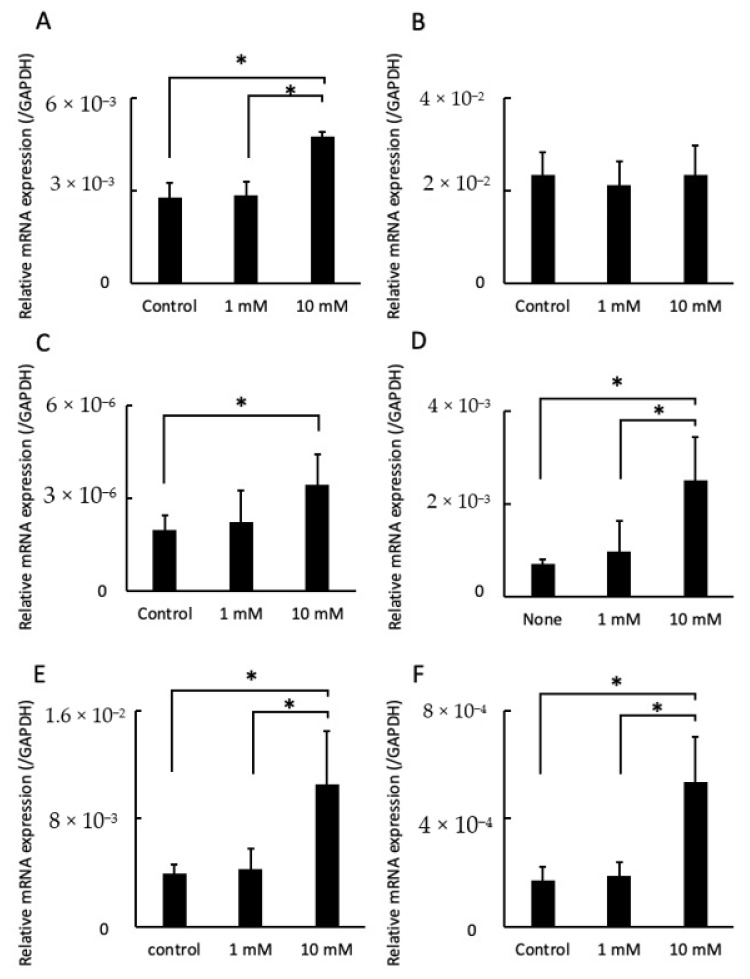
Effects of LA on mRNA expression levels of integrin α5 (**A**), integrin αV (**B**), IL-6 (**C**), IL-8 (**D**), ICAM-1 (**E**), and RANKL (**F**). The cells were cultured with fresh medium supplemented with 1 or 10 mM LA or fresh medium only (as a control). Total RNA was isolated from cells after culturing for 24 h. mRNA levels are shown relative to GAPDH, the internal control. * *p* < 0.05. Values are presented as the mean ± SD of six independent experiments.

Integrin α5 expression levels were greater in cells cultured with 10 mM LA than those treated with 0 mM (control) and 1 mM LA, while there was no significant difference in integrin αV expression levels (Figure 4A,B). IL-6 and IL-8 expression levels were significantly higher in cells treated with 10 mM LA than the control. In particular, IL-8 expression was about 3.5-fold greater than the control group and 2.6-fold greater than cells cultured with 1 mM LA (Figure 4C,D). ICAM-1 and RANKL expression levels were also significantly increased in cells cultured with 10 mM LA as compared to those treated with 0 mM (control) and 1 mM LA (Figure 4E,F).

## 3. Discussion

In this study, the effect of LA, a common metabolite of some oral bacteria, on PD was investigated. At first, scratch assay results showed that LA delayed wound healing of the junctional epithelium. Normally, the onset of PD involves proteases and other factors produced by periodontopathic bacteria that destroy the junctional epithelial cell layer and accelerate inflammation, which allows periodontopathic bacteria to invade tissues [29,30]. Therefore, from the results of this experiment, the delay in healing of the junctional epithelial cell layer due to the influence of LA may facilitate tissue invasion by oral bacteria and exacerbate PD. Although the cells were not destroyed by LA, proliferation was clearly suppressed, suggesting that a delayed cell cycle of junctional epithelial cells affected wound healing. Further cell cycle analysis revealed that a relatively high proportion of cells stimulated with a high concentration (10 mM) of LA were in the G0/G1 phase, indicating that LA arrested the cell cycle in the G0/G1 phase. This finding is similar to that of a previous study [13], which reported that butyric acid and propionic acid inhibit progression of the cell cycle of gingival fibroblasts. Although the time was delayed, the wound closed, suggesting that cell migration may have occurred; thus, the expression patterns of integrin α5 and αV, which are involved in wound healing (cell migration), were investigated. The results suggest that JE-1 cells produce integrin α5 to promote wound healing, although the addition of LA inhibited progression of the cell cycle, as evidenced by the relatively low density and number of cells. The small amount of total RNA isolated from cells stimulated with a high concentration of LA support this finding.

The results of qRT-PCR showed that the effect of LA may be linked to the progression of periodontitis because the expression of IL-6 was increased. It is well known that IL-6 promotes the differentiation of B cells into plasma cells, is involved in antibody production, and plays a central role in the inflammatory response. Furthermore, it has been reported that fibroblasts in gingival connective tissue produce IL-6 in response to calprotectin and may cause the progression of periodontitis via crosstalk between fibroblasts and macrophages [31]. Hence, IL-6 derived from the junctional epithelium might also contribute to this reaction. In addition, mRNA expression of the chemokine IL-8 was increased. IL-8 is an important factor in the regulation of the inflammatory response [32], promotes the migration of neutrophils from the peripheral blood to areas of inflammation in the gingival sulcus [33]. From the results of qRT-PCR that ICAM-1 expression was increased, the effect of LA on junctional epithelial cells may induce the migration of leukocytes into tissues and accelerate inflammation. ICAM-1 is an adhesive protein on the surface of the gingival and junctional epithelial cells that is an important component of adhesion between cells and the extracellular matrix, as well as cell signaling, inflammation, and immune responses in the development of periodontitis [34]. Increased mRNA and protein levels of ICAM-1 have also been observed in gingival epithelial cells stimulated with butyric acid [16]. However, further research is needed because the effects of cytokines vary from cell to tissue.

RANKL is the most important factor in osteoclast differentiation and function, is highly associated with alveolar bone loss in periodontal lesions [35].In addition, RANKL, which promotes osteoclast formation, is produced by osteoblasts, osteocytes, periodontal ligament cells, and gingival epithelial cells of the periodontium [36]. Interestingly, LA significantly increased mRNA expression levels of RANKL. Therefore, a very important finding of this study was that a high concentration of LA could accelerate alveolar bone loss. However, since this was an in vitro study, it is unclear whether the same effect will occur in vivo. More experiments that are in line with actual clinical practice using a mouse model are required.

The results of the present study suggest that LA produced by bacteria in subgingival plaques suppresses cell proliferation and delays wound healing by inhibiting the cell cycle of JE-1 cells at high concentrations. Further, stimulation of the junctional epithelium with a high concentration of LA exacerbates inflammation via excessive production of inflammatory cytokines and adhesion factors and could be directly involved in alveolar bone loss. Therefore, LA, like butyric acid, has a positive effect on human health but can also have a harmful effect locally such as the promotion of PD. The effects of LA on the junctional epithelium revealed in this study may help clarify some of the onset and progression of PD and may suggest new treatments and preventions.

## 4. Materials and Methods

### 4.1. Cell Treatment

Murine junctional epithelial (JE-1) cells, which were established by Matsuo Yamamoto (Showa University, Tokyo, Japan [28]), were cultured in CnT-Prime epithelial culture medium (CELLnTEC Advanced Cell Systems AG, Bern, Switzerland) under a humidified atmosphere of 5% CO_2_/95% air at 37 °C. At 80% confluence, the cells were detached from the culture dishes using TrypLE™ recombinant enzyme (Gibco, Carlsbad, CA, USA).

### 4.2. Scratch Assay

JE-1 cells were grown in the wells of 24-well cell culture plates until 100% confluence. Then, the cell monolayer was scratched using a pipette tip, washed with phosphate-buffered saline to remove floating cells, and incubated in CnT-Prime medium supplemented with 1 or 10 mM LA or fresh medium only (as a control). Finally, the cells were photographed on days 0, 1, and 2 using an inverted stage microscope (BZ-9000; Keyence, Osaka, Japan) equipped with a digital camera at 100× magnification. The cell-free area was calculated using ImageJ software 1.53k (https://imagej.net/ (accessed on 1 September 2021)).

### 4.3. RNA Extraction

The cells were seeded into the wells of 24-well cell culture plates at 7.0 × 10^4^ cell/well in CnT-Prime medium and cultured for 48 h. The medium was changed to either fresh medium supplemented with 1 or 10 mM LA or fresh medium only (as a control). Following incubation for 24 and 48 h, total RNA was extracted from the cells using an RNeassy Mini Kit (Qiagen GmbH, Hilden, Germany) in accordance with the manufacturer’s instructions. The RNA concentration was then measured with a NanoDrop™ Lite spectrophotometer (NanoDrop Technologies, LLC, Wilmington, DE, USA).

### 4.4. Cell Proliferation Assay

Cell proliferation was analyzed using the Cell Counting Kit-8 (Wako, Osaka, Japan) in accordance with the manufacturer’s instructions. The cells were plated into the wells of 96-well cell culture plates at 5 × 10^3^ cell/well in CnT-Prime medium and incubated for 24 h. Afterward, the medium was changed to fresh medium supplemented with 1 or 10 mM LA or fresh medium only (as a control). The absorbance was recorded every 24 h for 4 days (including day 0) with a SpectraMax^®^ M2 microplate reader (Molecular Devices, LLC, San Jose, CA, USA). The optical density of the wells was measured at a wavelength of 450 nm with a reference wavelength of 600 nm.

### 4.5. Cell Viability Assay

Cell viability was analyzed using the lactate dehydrogenase (LDH) Cytotoxicity Detection Kit (TaKaRa Bio, Inc., Shiga, Japan) in accordance with the manufacturer’s instructions. The cells were incubated in the wells of 24-well cell culture plates at 7.0 × 10^4^ cell/well in CnT-Prime medium. At 24 and 48 h, the supernatants were collected and treated with 1% Triton X 100 prior to measurement of maximum enzyme activity. Absorbance was measured using a SpectraMax^®^ M2 microplate reader (Molecular Devices, LLC.). The optical density of the wells was measured at a wavelength of 490 nm with a reference wavelength of 600 nm.

### 4.6. Cell Cycle Assay

The phase of the cell cycle was measured using the Cell-Clock Cell Cycle Assay Kit (Biocolor Ltd., Carrickfergus, Northern Ireland) in accordance with the manufacturer’s instructions. The cells were pre-cultured in the wells of 24-well cell culture plates at 5 × 10^4^ cell/well in CnT-Prime medium for 24 h. Afterward, the medium was changed to fresh medium supplemented with 1 or 10 mM LA or fresh medium only (as a control). Then, live cells were labeled with the dye reagent of the Cell-Clock Cell Cycle Assay Kit at 24 and 48 h. Images were obtained with an inverted stage microscope (Dmi1; Leica Camera AG, Wetzlar, Germany) equipped with a digital camera at 100× magnification. The phase of the cell cycle was determined by color pixel analysis using ImageJ software 1.53k (https://imagej.net/ (accessed on 1 September 2021)).

### 4.7. Real-Time Quantitative Reverse-Transcription Polymerase Chain Reaction (qRT-PCR)

Total RNA was extracted from cells cultured in fresh medium supplemented with 1 or 10 mM LA or fresh medium only (as a control) for 24 h, reverse transcribed into cDNA using PrimeScript RT Master Mix (TaKaRa bio, Inc.) with TB Green Premix Ex Taq Ⅱ polymerase (TaKaRa Bio, Inc.) and amplified with a Thermal Cycler Dice Real-Time System (TaKaRa Bio, Inc.) using primer pairs specific for integrin α5, integrin αV, interleukin (IL)-6, IL-8, intercellular adhesion molecule-1 (ICAM-1), receptor activator of nuclear factor kappa-B ligand (RANKL), and glyceraldehyde-3-phosphate dehydrogenase (GAPDH), as an internal control. Gene expression levels were normalized to that of GAPDH. The qRT-PCR cycling conditions consisted of an initial denaturation step at 95 °C for 30 s followed by 40 cycles of denaturation at 95 °C for 5 s and annealing and extension at 60 °C for 30 s. Melting curve (dissociation curve) analysis was performed to confirm the specificity of primers.

### 4.8. Statistical Analysis

Data are presented as the mean ± standard deviation (SD). Statistical analyses were conducted with MacTKV3 software (Esumi Co., Ltd., Tokyo, Japan). The Shapiro–Wilk test was used to confirm the normality of the data (Figure 2, Figure 3 and Figure 4), while the Bartlett’s test was performed to assess homoscedasticity (Figure 2, Figure 3 and Figure 4). For groups with homoscedasticity, Bonferroni’s multiple comparison test was used after one-way analysis of variance (Figure 2, Figure 3 and Figure 4A–C). For groups with heteroscedasticity, the Games–Howell multiple comparison test was used after Welch’s analysis of variance (Figure 4D–F). A probability (*p*) value of < 0.05 was considered statistically significant.

## Data Availability

All the data obtained in this research are described in the manuscript.
